# Evaluating, Filtering and Clustering Genetic Disease Cohorts Based on Human Phenotype Ontology Data with Cohort Analyzer

**DOI:** 10.3390/jpm11080730

**Published:** 2021-07-27

**Authors:** Elena Rojano, José Córdoba-Caballero, Fernando M. Jabato, Diana Gallego, Mercedes Serrano, Belén Pérez, Álvaro Parés-Aguilar, James R. Perkins, Juan A. G. Ranea, Pedro Seoane-Zonjic

**Affiliations:** 1 Department of Molecular Biology and Biochemistry, University of Málaga, 29071 Málaga, Spain; elenarojano@uma.es (E.R.); josecordoba@uma.es (J.C.-C.); apareslar@gmail.com (Á.P.-A.); ranea@uma.es (J.A.G.R.); seoanezonjic@uma.es (P.S.-Z.); 2 Institute of Biomedical Research in Málaga (IBIMA), 29010 Málaga, Spain; jabato@uma.es; 3 Supercomputation and Bioinformatics (SCBI), University of Malaga, 29071 Malaga, Spain; 4 LifeWatch-ERIC, 41071 Seville, Spain; 5 Centro de Investigación Biomédica en Red de Enfermedades Raras (CIBERER), [Madrid, Málaga, Barcelona], Instituto de Salud Carlos III, 28029 Madrid, Spain; dgallego@cbm.csic.es (D.G.); mserrano@sjdhospitalbarcelona.org (M.S.); bperez@cbm.csic.es (B.P.); 6 Centro de Diagnóstico de Enfermedades Moleculares, Centro de Biología Molecular-SO UAM-CSIC, Campus de Cantoblanco, Universidad Autónoma de Madrid, 28049 Madrid, Spain; 7 Instituto de Investigación Sanitaria idiPAZ, 28049 Madrid, Spain; 8 Neuropediatric Department, Institut de Recerca Hospital Sant Joan de Déu, 08950 Barcelona, Spain

**Keywords:** genetic diseases, cohort analyzer, human phenotype ontology, cluster analysis, phenotype quality assessment

## Abstract

Exhaustive and comprehensive analysis of pathological traits is essential to understanding genetic diseases, performing precise diagnosis and prescribing personalized treatments. It is particularly important for disease cohorts, as thoroughly detailed phenotypic profiles allow patients to be compared and contrasted. However, many disease cohorts contain patients that have been ascribed low numbers of very general and relatively uninformative phenotypes. We present Cohort Analyzer, a tool that measures the phenotyping quality of patient cohorts. It calculates multiple statistics to give a general overview of the cohort status in terms of the depth and breadth of phenotyping, allowing us to detect less well-phenotyped patients for re-examining or excluding from further analyses. In addition, it performs clustering analysis to find subgroups of patients that share similar phenotypic profiles. We used it to analyse three cohorts of genetic diseases patients with very different properties. We found that cohorts with the most specific and complete phenotypic characterization give more potential insights into the disease than those that were less deeply characterised by forming more informative clusters. For two of the cohorts, we also analysed genomic data related to the patients, and linked the genomic data to the patient-subgroups by mapping shared variants to genes and functions. The work highlights the need for improved phenotyping in this era of personalized medicine. The tool itself is freely available alongside a workflow to allow the analyses shown in this work to be applied to other datasets.

## 1. Introduction

Advances in genome sequencing and bioinformatics analysis have led to their widespread usage in genetic disease diagnosis [1,2,3]. These technologies can be used to better understand functional genomic elements and how variants can lead to disease [4,5]. They have been applied to multiple diseases, including cardiovascular diseases [6] and cancer [7], and used to identify risk factors for severe COVID-19 [8]. They can also be used to improve our knowledge of rare diseases.

However, to extract meaning, genome sequencing must be accompanied by the complete and accurate clinical characterization of patients. Multiple resources are available to ascribe phenotypes using clinical terminology [9], such as the Human Phenotype Ontology (HPO) [10], a standardized vocabulary of hierarchically organized terms. It is widely used by tools for clinical diagnosis, such as ClinPhen [11], Phenomizer [12] and Phenotips [13]. It is also used to annotate the pathological traits of patients and describe diseases in resources such as DECIPHER [14], Orphanet [15] and Monarch [16].

Despite the range of tools available to aid patient characterization, in many cases, this information is incomplete. This may be due to reasons such as the complexity of a patient phenotypic profile or insufficient consultation time to obtain full diagnosis. This is a problem, as whilst a cohort of patients diagnosed with the same disease often share common pathological phenotypes, other phenotypes may be specific to individuals. As such, treatments may work for one patient but not for another, potentially even aggravating their symptoms. Cohorts can also contain subgroups of similar patients. Identifying them is key to improving diagnosis [17].

Another problem is the level of precision used when characterizing patients. For accurate diagnosis and to better understand disease, patients should be described in the most specific terms possible. Precision medicine should also be applied to the clinics, not just to molecular and genetic medicine. This is especially important when investigating cohorts, as it allows patients to be compared accurately.

In previous work from our group, we applied various network-based techniques to cohorts of phenotyped patients [18,19,20,21]. However, despite finding interesting results, the phenotypic data corresponding to many of the patients did not contribute to the analysis, due to low specificity, showing that highly specific and precise phenotyping is crucial to make sense of large-scale cohort data [18,22,23].

Despite the importance of patient phenotyping to better understand and diagnose genetic diseases, there are currently no publicly available resources to evaluate phenotype information in a patient cohort. Motivated by this, we developed Cohort Analyzer, a software tool to analyse a cohort of patients annotated with HPO terms. For this, it calculates multiple summary statistics for the entire dataset, produces plots of the term frequency distribution across the levels of the HPO, measures information content (IC) for each patient profile and more. Patients can be compared and clustered based on phenotypic similarity. If available, it can also assess genetic variant data in terms of coverage analysis. It produces HTML reports, allowing a researcher to assess the information available within a given cohort. Code is available as part of the Patient Exploration Tools Suite (https://github.com/ElenaRojano/pets) [23].

Here, we apply Cohort Analyzer to three datasets: the DECIPHER database encompassing many thousands of patients, obtained from many different centres and countries with complex and heterogeneous genetic diseases [14], data from a national initiative that includes patients with a specific disease that is loosely defined as including intellectual disability/developmental delay (ID/DD) and/or multiple congenital anomalies (MCA) [24], and a relatively small cohort of patients with a monogenic disease, PMM2-CDG (MIM# 212065), characterized by a group of specialized experts. These analyses provide multiple insights into the information available from each dataset and help guide further analysis, such as whether to filter low-information patients, obtain subgroups of phenotypically similar patients, and connect phenotype information with genotype, genes and functions.

## 2. Materials and Methods

### 2.1. Cohort Analyzer

Cohort Analyzer has been designed to analyse the phenotypic information available within a patient disease cohort. Phenotypes are defined using the HPO, a hierarchical classification of standardized human pathological traits [25]. It can also analyse genomic data for the same cohort if available.

To evaluate the phenotypic status of a cohort, Cohort Analyzer calculates summary statistics related to HPO term frequency and diversity, analyses where the terms lie among the different levels of the HPO to determine how deeply the patients have been phenotyped, and uses information content (IC) to assess whether the terms tend to be informative or not. In addition, it performs two different cluster analyses: (1) Naïve clustering, to assess whether the dataset contains groups of patients for which little phenotypic information is available; and (2) Semantic clustering, to detect groups of phenotypically similar patients, using a clustering method that incorporates semantic similarity measures. To evaluate genomic data quality, Cohort Analyzer calculates additional summary statistics related to the properties of the variants and performs genome coverage analysis.

Cohort Analyzer produces HTML reports, a Main Report and Clustering Reports, including multiple tables and graphics to aid results interpretation. It forms part of the Patient Exploration Tool Suite (PETS) [23], developed in Ruby and available from https://github.com/ElenaRojano/pets. To facilitate the use of Cohort Analyzer, and recreate the analyses as applied to the cohorts presented here, we have developed a workflow, available from https://github.com/JoseCorCab/cohortAnalyzer_wf. This workflow provides full instructions to allow the interested user to apply Cohort Analyzer to their own dataset.

#### 2.1.1. General Statistics Calculation

Cohort Analyzer calculates multiple summary statistics to give an overview of the cohort in terms of phenotyping breadth and depth as described in Table 1. Summary statistics are also calculated for the genomic data if available.

#### 2.1.2. Human Phenotype Terms Frequency and Distribution Analysis

The frequency of each HPO term in the cohort, defined as the number of patients suffering a given phenotype, divided by the total number of patients, is calculated and shown in the Main Report in the searchable table “HPO stats”.

To further investigate phenotyping specificity, the depths of these terms within the hierarchical structure of the HPO are also analyzed. For each term, depth is calculated as the shortest path from the HPO root node. Values can range from 1 (root node—the most general term in the HPO) to 16 (the deepest and thus most specific node). Depth can be calculated taking into account how often the term occurs within a cohort (“weighted cohort”), or using the unique set of terms (“unique terms cohort”).

By comparing the depth of the terms in the cohort to the depth of all terms within the HPO, we can assess whether the patients in the cohort tend to be phenotyped using more or less specific terms. The relative and absolute distributions of the HPO terms levels in the cohort are shown in the Main Report, in the section called “HPO annotations distribution”, alongside the distribution of levels for all HPO terms within the HPO.

#### 2.1.3. Dataset Specificity Index

**Figure 1:**
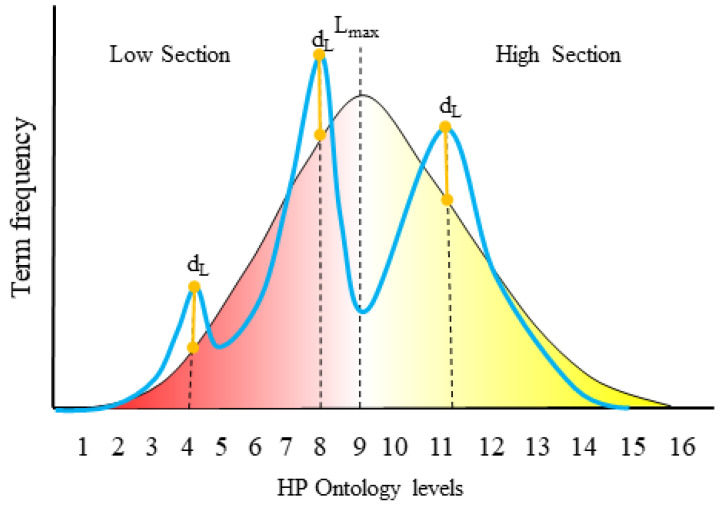
Term level distribution for the HPO (black line) and for a hypothetical patient cohort (blue line). The levels in the ontology are divided in two sections, the *Low section* and the *High section*. The reason for the division is based on the probability of a level being used, which is related to the number of levels that separate it from the root node in the ontology, and the number of terms contained at the level. $L_{max}$ is the level with largest number of terms in the ontology and it is the last level for which deeper phenotyping leads to a greater number of possible terms. The following levels have decreasing term counts, thus they have smaller probabilities to appear in a patient profile. By dividing the ontology into sections, we can see how a real patient cohort uses the ontology by measuring the distribution differences at each level ($d_{L}$). These differences are weighted taking into account the sections and how many terms belong to each level, and are used to calculate the DsI to measure the phenotype information of a patient cohort.

To assess HPO term specificity within a cohort with respect to the HPO hierarchy, we calculate the Dataset specificity Index (DsI). First, we divide the HPO into two sections, *Low section*: the ontology levels from root to the level with the highest number of terms ($L_{max}$), *High section* the ontology levels from $L_{max}$ to the deepest term in the ontology (Figure 1).

As such, given a set of HPO terms from a cohort, we penalise terms at shallower levels of the hierarchy, and reward terms at deeper levels. Moreover, we can increase the penalty/reward in relation to how many HPO levels a given term is from $L_{max}$, the most popular level in the ontology, under the rationale that HPO terms that are close to the root in the ontology will be reached easily and have multiple child terms, defined as terms within the ontology that are descendants of a given term and thus represent more specific phenotypes. Conversely, the very deep terms are more difficult to reach and give much more specific information.

More formally, we can compute scores for both sections in the following manner:

Firstly, for each level within the hierarchy, a difference score, $d_{L}$, is calculated (Equation (1)):

(1) $$d_{L}=Pobs_{L}-Pont_{L}$$

This represents the difference between the proportion of terms observed in the cohort ($Pobs_{L}$) and the proportion of terms in the ontology ($Pont_{L}$) for a given level *L*.

The different scores are then used to calculate overall scores for the two sections. Only $d_{L}$ scores greater than 0 will contribute to a section score.

The overall Low section Score, (LsS, Equation (2)) is calculated using the following:

(2) $$LsS=\frac{\sum_{L=1}^{L_{max}}d_{L}*\left(L_{max}-L+1\right),\;\mathrm{if}\;d_{L}>0}{L_{S}}$$

This shows that the $d_{L}$ scores for the *Low section* of the ontology are weighted, where the weights are assigned inversely to the level of the ontology. $L_{max}$ is the level with the highest number of terms in the ontology. The weights serve to penalize very unspecific terms in the cohort dataset. $L_{S}$ is the number of levels present in the ontology section.

The overall High section Score (HsS, Equation (3)) is calculated using the following:

(3) $$HsS=\frac{\sum_{L=L_{max}+1}^{L_{o}}\left(d_{L}*\left(L_{o}-L\right)\right),\;\mathrm{if}\;d_{L}>0}{L_{S}}$$

This shows that the $d_{L}$ scores for the *High section* of the ontology are weighted, where each level is weighted in proportion to its depth with respect to $L_{max}$. The weights serve to reward very specific terms in the cohort dataset. $L_{o}$ is the deepest possible level in the ontology.

Finally, the Dataset specificity Index is the ratio between both section scores (Equation (4)):

(4) DsI=HsS/LsS

The *DsI* will be 0 if the *HsS* is 0 and infinite if the *LsS* is 0. A value of 1 means that the contribution of both sections are the same; however, in practice, *DsI* values tend to be below 1 because of the difficulty of reaching the *High section* levels.

#### 2.1.4. Cluster Analysis and Information Content Distribution

Cohort Analyzer uses the phenotype profiles of each patient to perform cluster analysis. Initially, Naïve clustering is performed, in which patient HPO term profiles are represented as binary vectors, without taking into account the relationship between terms. Euclidean distance between the vectors is calculated and clustering is performed using Ward’s method [26]. To visualize the clustering results, boxplots are shown for the largest clusters, with the number of patients in each cluster also indicated in Main Report section “Clustering patients by HPO profile”. The boxplots show the average information content (IC) for the HPO terms assigned to the patients in each cluster, where IC is defined as $-log_{10}$ of the HPO term frequency [25]. Furthermore, the IC distribution per cluster is complemented with the patient profile size distribution. This procedure can be used to assess whether the cohort contains large groups of uninformative patients.

In addition, Cohort Analyzer provides plots comparing the IC of the HPO terms in the cohort in relation to the ontology (“HPO ICs distribution”). In this approach, we used IC to indicate terms specificity [10]: IC values <1 represent unspecific HPO terms. It is also performed at the patient level, by calculating the average IC for all HPO terms assigned to each patient, for both the cohort-inferred IC and ontology-inferred IC values.

A second clustering is performed to compare the patients based on the similarity of their profiles, using semantic similarity measures that take into account HPO hierarchical structure. Cohort Analyzer can use three such measures, Resnik, Lin and Jiang-Conrath [27]. For the analysis of the datasets presented here, we use Lin method, which produces similarity scores normalized between 0 and 1. The scores are used to produce a dissimilarity matrix, which is hierarchically clustered. The groups of patients are then obtained using the cutreeDynamic function from the R package dynamicTreeCut (Version 1.63-1) [28], with the parameter minClusterSize set to one hundredth of the total number of patients in the cohort (or two if this is higher).

Clustering results are provided as heatmaps in the Main Report, that represent the profile similarity between patients and the clustering partitioning. Then, for each semantic measure method, a Clustering Report is created, including a table (Patient HPO profiles by cluster) with information on the patients in each cluster, including the HPO codes with links to the HPO website (https://hpo.jax.org, accessed on 14 April 2021) and the full phenotype names.

### 2.2. Case Studies: Assessing the Phenotypic and Functional Space within the Top Patient Clusters

Average similarity was calculated between the patients for each cluster produced by the semantic clustering method. The top clusters in terms of similarity were further studied to investigate the diversity of phenotypes for the DECIPHER and ID/MCA datasets. For each of these clusters, the phenotypic spectra were obtained, aggregating all the HPO terms within the cluster and removing the parental terms. The phenotype profile of each patient was compared to this spectra, taking into account semantic similarity. For each patient, an individual semantic similarity is computed for each combination of HPO term in the patient profile and HPO term in the phenotypic spectra. For each term in the patient profile, its highest match with the phenotypic profile is selected. These results are displayed in the form of a heatmap. Patients are shown on the x-axis, sorted by decreasing semantic similarity with the phenotypic spectra. Phenotypes are shown on the y-axis, ordered by the average similarity values across all patients. The cells of the heatmap represent the semantic similarity between a patient term and a phenotypic spectra term. Up to 20 HPO terms and 40 patients are shown.

In addition, functional enrichment analysis was performed for the genes mapping to the patient variants. For the ID/MCA dataset, genes corresponding to variants shared by at least two patients were used; for DECIPHER dataset the genes were shared by at least four patients, motivated by the differing cluster sizes. Enrichment was performed using clusterProfiler [29] for Gene Ontology (GO) terms [30]. Results are shown as Enrichment Maps.

### 2.3. Cohort Descriptions

We used three different cohorts of patients to conduct this study, all of which had been phenotypically annotated using HPO terms. The latest version of this ontology (April 2021 release) includes more than 13,000 different terms. General characteristics of each dataset are given in Table 2.

#### 2.3.1. DECIPHER Cohort

The information for this cohort was downloaded from the DECIPHER database (version 2021-04-28, mapped to the GRCh38/hg38 human genome assembly), under the DECIPHER consortium Data Access Agreement [14]. This version includes 30,436 patient records, of which we selected 22,018 with annotated pathological phenotypes. This cohort also includes copy-number variant (CNV) coordinates from microarray-based Comparative Genomic Hybridization (aCGH).

#### 2.3.2. ID/MCA Cohort

This cohort is derived from a study evaluating the contribution of de novo and inherited CNVs to phenotypes related to intellectual disability/developmental delay (ID/DD) occurring alongside multiple congenital anomalies (MCA) [24]. From this study, we obtained phenotype information for 4183 patients and genotype information for 1027 included in the study, available in the Supplementary Material (Table S7. Phenotype data of patients included in this study) from [24]. Genome coordinates are under the NCBI36/hg18 human genome assembly.

#### 2.3.3. PMM2-CDG Cohort

This cohort contains HPO annotated pathological phenotype and genotypic information for 27 patients suffering from phosphomannomutase 2 congenital disorder of glycosylation (PMM2-CDG), including terms related to their neurological, multisystem and dysmorphologic features [31,32,33]. All patients had variants in the locus (chr16:8891670-8943194), corresponding to the *PMM2* gene coordinates in the GRCh37/hg19 human genome assembly. The study was approved by the Ethics Committee of the Universidad Autónoma de Madrid (CEI-105-2052) and conducted according to the principles of the Declaration of Helsinki. All participants gave informed consent.

## 3. Results

### 3.1. Revealing Differences between Cohorts in Terms of Phenotype Information

We applied Cohort Analyzer to the three different patient cohorts to show how it can be used to investigate phenotype information in datasets of very different designs.

As shown in Table 3, the DECIPHER dataset shows a much larger number of unique HPO terms and patients than the others, as expected given the resource was designed to collect data for a wide range of phenotypically heterogeneous patients as part of an international initiative [14]. The ID/MCA and PMM2-CDG datasets show much closer numbers of unique HPO terms; however, they differ greatly in terms of the number of patients. The average numbers of HPO terms per patient differ greatly between groups, being several times higher for the PMM2-CDG dataset. Even more striking is the difference in terms of the number of phenotypes at the 90th percentile, indicating that 10% of DECIPHER and ID/MCA patients have only one HPO term to define their clinical profile in contrast to PMM2-CDG cohort, for which 90th percentile patients have 15 HPO terms.

In terms of phenotype depth, for most of the HPO terms used to describe the patients in the ID/MCA dataset, more specific child terms were available. This also occurred with the DECIPHER and PMM2-CDG datasets but to a much lesser extent, in fact almost half of the HPO terms used in the PMM2-CDG dataset were the most specific terms available. Furthermore, the patient profile length in PMM2-CDG dataset is very large, ~5 times and ~10 times the size of DECIPHER and ID/MCA datasets, respectively. Of particular note was that the ID/MCA cohort patients were assigned less than three phenotypes on average, and that almost all of the phenotypes had more specific ancestors.

These summary statistics provide a clear overview of the properties of the different datasets in terms of how thoroughly the patients have been phenotyped in terms of both breadth and depth; moreover, they give an idea of how consistent the phenotyping is across patients.

**Figure 2:**
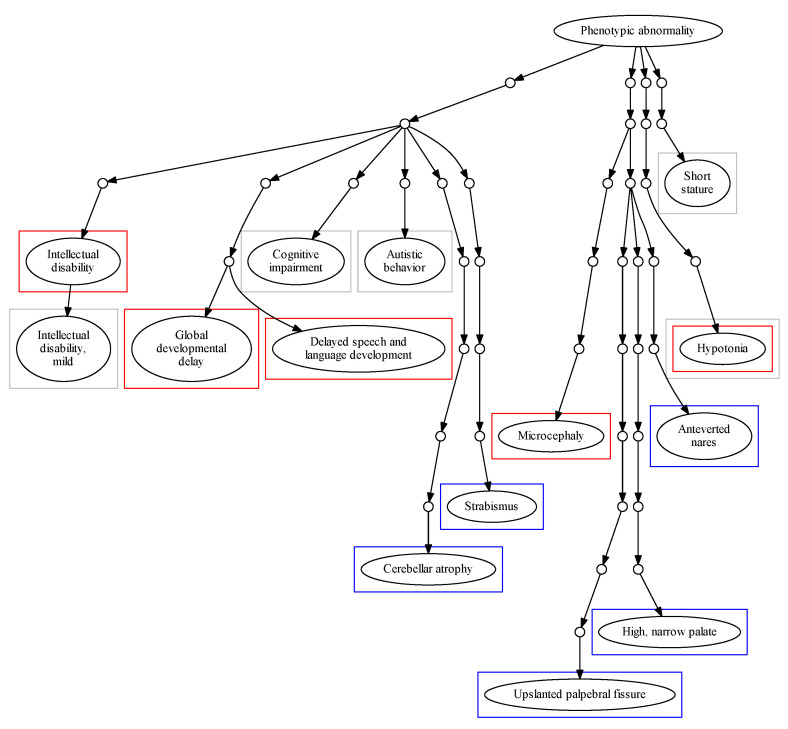
Schematic representation of top five most frequent HPO terms for each cohort in relation to the “Phenotypic abnormality” node. Coloured squares represent terms for each cohort. Red: DECIPHER. Gray: ID/MCA. Blue: PMM2-CDG.

Cohort Analyzer can be used to assess the most frequent HPO terms among patients within a dataset. It was applied to the three datasets used in this study (Table 4). Figure 2 shows the position of these phenotypes within the HPO hierarchical structure. The term HP: “Intellectual disability” and its degrees were highly frequent among patients in the DECIPHER and ID/MCA datasets, with 34.98% of patients in DECIPHER dataset being ascribed this term, and 17.67% of patients in the ID/MCA cohort ascribed its child term, HP: “Intellectual disability, mild”. Whilst the high prevalence of such a term in these datasets might be expected, and it may be a useful phenotype in conjunction with a highly detailed phenotypic profile, by itself it is less useful; moreover, it is found at level 5 of the HPO and its child terms only describe different severity grades; as such, it represents somewhat of a phenotyping dead end within the HPO. This pathological trait is complex and encompasses multiple cognitive deficits expressed to several degrees, with multiple potential causes. Therefore, its precise description can be overly diffuse [34]. Similar problems occur with other frequent, general terms, such as HP: “Global developmental delay”. This limits the ability of the practitioner to provide a more specific diagnosis in this branch of the HPO. For other phenotype, such as HP: “Cognitive impairment”, ascribed to 80.96% of the ID/MCA cohort members, there are myriad child terms available, including HP: “Mental deterioration” and HP: “Memory impairment”, suggesting unexplored phenotypic space within the cohort. This is also the case for HP: “Delayed speech and language development”, which is at the sixth HPO level but has several child levels.

For the PMM2-CDG cohort, we found significant differences between the top terms of this dataset in comparison with the DECIPHER and ID/MCA datasets. We found that all patients were described with HP: “Cerebellar atrophy” and most of them with HP: “Upslanted palpebral fissure” (88.88%), two very specific terms (ninth and eleventh HPO levels, respectively). In fact, the frequency of these pathological terms within the cohort is very high, revealing a high level of phenotypic homogeneity in the cohort.

This characteristic could be expected for a monogenic disease dataset. However, it is interesting that the specific HPO terms describing precise attributes of PMM2-CDG dataset are the most prevalent, whereas for the other cohorts the most common terms are far more general. This makes sense given the findings in Table 3, showing that many of the phenotypes ascribed to these patients are the most specific possible within the HPO, i.e., have no child terms.

**Figure 3:**
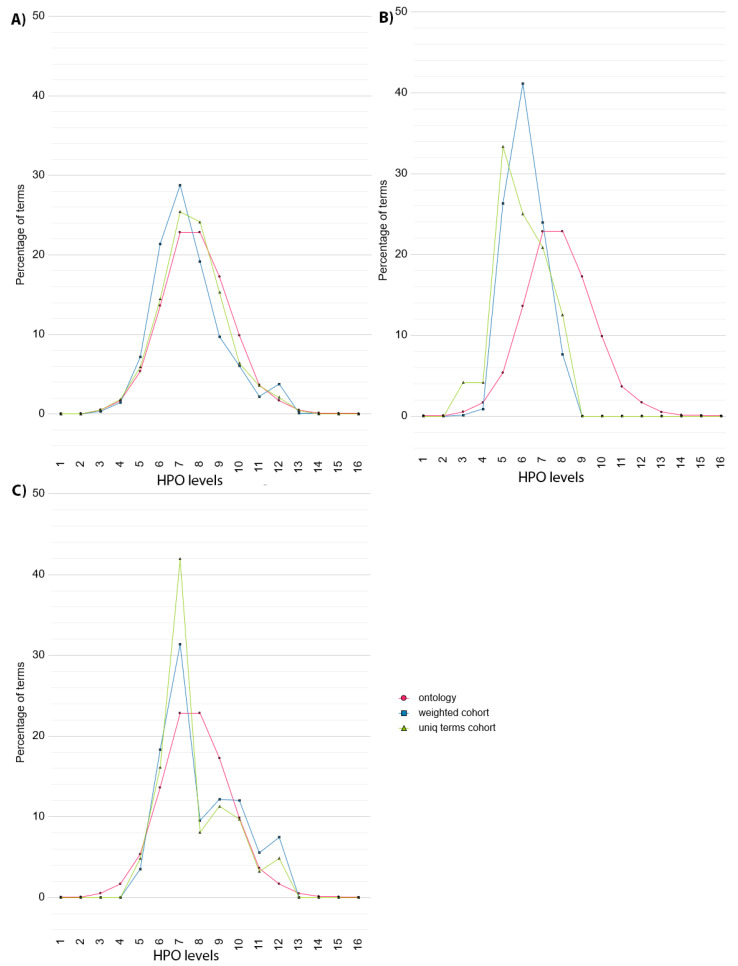
HPO term percentage distribution plots for (**A**) DECIPHER, (**B**) ID/MCA and (**C**) PMM2-CDG datasets. Green curves represent the percentage of unique HPO terms used to describe the cohort, blue curves take into account the frequency of each term and pink curves show the percentage of terms included in the HPO at each level.

To provide a more detailed overview of phenotype specificity, Cohort Analyzer compares the distribution of HPO term levels used within a given cohort to the distribution of HPO term levels for all terms within the ontology (Figure 3). This is performed taking into account term frequency (blue curves, “weighted cohort”) or counting each unique term only once (green curves, “unique terms cohort”). This distinction is important as a single, highly phenotyped patient could strongly affect the unique terms cohort, but its effect on the weighted cohort curve would be diluted. The distribution of terms within the HPO is represented as a pink curve.

In the case of DECIPHER dataset (Figure 3A), the HPO terms used in the dataset (green curve) show a similar distribution to the HPO (pink curve), with two peaks at level 7 and 8. When considering the frequency of each term (blue curve), the distribution is shifted slightly towards the initial levels of the HPO, although there is a small increase compared to the HPO at level 12.

In contrast, the distribution shown by the ID/MCA cohort data (Figure 3B) are skewed far more to the left, towards the initial levels of the HPO (green curve), with peaks at level 3 and 5. There are no terms described from level 8 onwards, showing that the deepest half the HPO has not been used to describe the patients, suggesting unexplored phenotypic space for this dataset.

For the PMM2-CDG dataset, the distribution of unique HPO terms (green curve) has a small increase at level 6 and a high peak at level 7, followed by a smaller peak at level 12. When the HPO term frequency is considered (blue curve), this shifts in favour of deeper levels of the HPO, reducing the high peak at level 7 and increasing the peaks at 10 to 12. This pattern is suggestive of a common phenotype at level 7, but additional, more specific phenotypes at deeper levels. The shift to the right when taking term frequency into account suggests that many of the patients have been phenotyped deeply.

To quantify the extent to which a cohort has been phenotyped in terms of HPO depth, we used the Dataset specificity Index (DsI), applying it to all cohort datasets, both for unique terms and considering term frequency (Table 5).

In the case of DECIPHER dataset, the DsI value is 0.13 for the unique HPO terms used to describe the cohort, in accordance to the distribution shift to the shallower levels of the HPO observed in Figure 3A. When DsI is computed taking the frequency of each term within the cohort into account, the value slightly increases to 0.195, due to the peak at level 12. This suggests that DECIPHER patients are described using a wide range of HPO terms, representative of the HPO itself, in line with the nature of the resource. However, when we consider term frequency, the reduction in DsI suggests that many patients are actually phenotyped using much less specific terms.

In the case of ID/MCA cohort, DsI values for both unique HPO terms and the frequency of each term within the cohort is zero because this dataset has zero phenotypes in the *High section* of the HPO, in line with Figure 3B.

Higher DsI values where found for the PMM2-CDG dataset. Considering the unique HPO terms used to describe the cohort, the DsI value was 0.27; however, when calculating the frequency of each term within the cohort, this increased to 1.06. This increase in score suggests that many of the patients have been deeply phenotyped, in line with the change in distributions seen in Figure 3B—peaks at levels 10, 11 and 12 explain this increment. Again, this suggests that not only are highly informative phenotypes used for this dataset, they have been used to described a relatively large number of patients.

**Figure 4:**
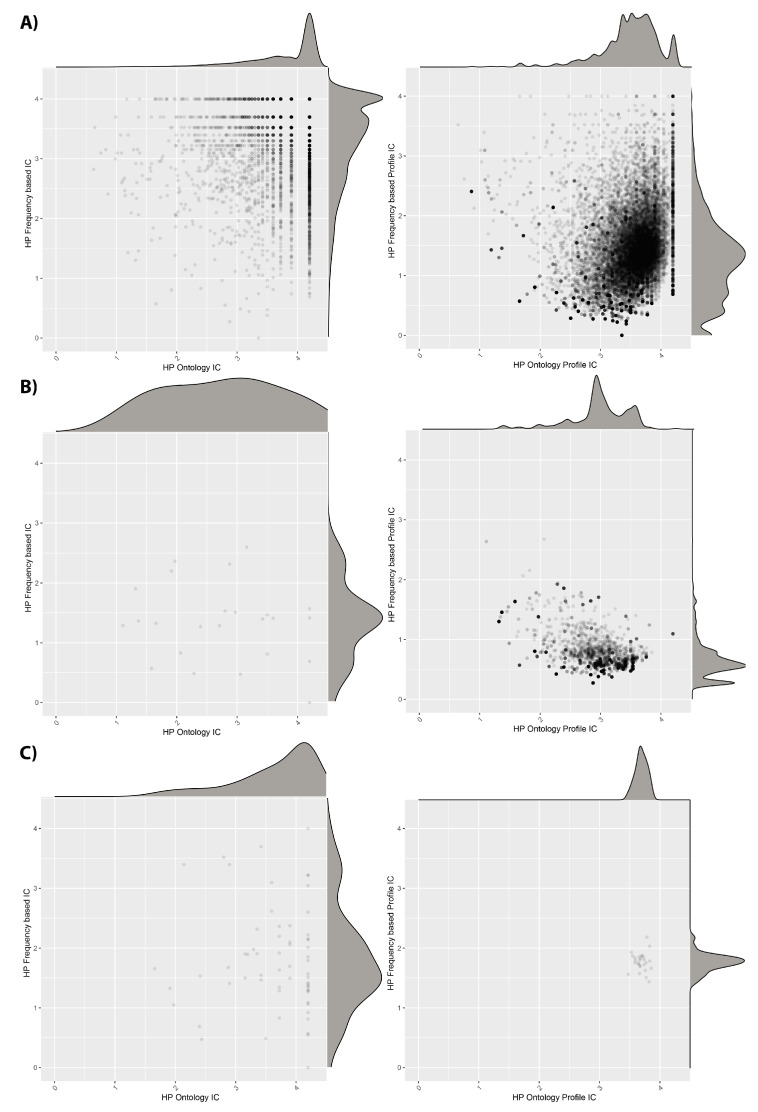
Information content (IC) distribution for (**A**) DECIPHER, (**B**) ID/MCA and (**C**) PMM2-CDG datasets. Left figures correspond to HPO terms and right figures to patient phenotype profiles. The ”Frequency based IC” values were computed using all patients from the three cohorts to make a coherent comparison.

The information content (IC) values for individual HPO terms and phenotypic profiles, in terms of their frequency within the HPO and the cohorts, are shown in Figure 4. We see that the DECIPHER dataset uses HPO terms with relatively high IC according to both the ontology and the dataset calculations. However, when we look at the IC averaged across patient profiles, the dataset-frequency IC drops dramatically. This suggests that, whilst there are many informative HPO terms used in DECIPHER, the majority of the patients have combinations of less specific ones, in line with the reduction in DsI shown in Table 5 between unique and weighted values. For the ID/MCA dataset, the individual ICs are less informative, as is also the case for the patient profile ICs, also in line with Table 5. However, in the case of the PMM2-CDG dataset, although the individual ICs are quite low, when IC is calculated for the patient profiles, it improves, leading to higher values than for the other datasets. This also fits with the DsI values, and fits with the idea that the patients within this dataset have been consistently phenotyped to a deep level.

### 3.2. Identifying Patient Subgroups with Low Information Profiles

Cohort Analyzer also performs clustering analysis to assess the phenotypic information in a cohort and identify patients with less informative phenotypic profiles. This initial procedure ignores the ontological attributes of the HPO terms; as such, we have named it Naïve clustering. We assume that if patients within a cohort are well-phenotyped, their profiles will include multiple, specific HPO terms. Conversely, the profiles of uninformative patients will include smaller profiles with more general HPO terms. As such, the profiles of these patients are more likely to be similar across a cohort and, therefore, to cluster together.

**Figure 5:**
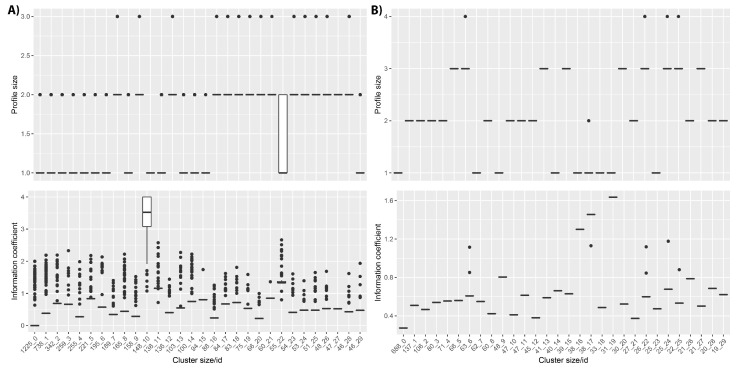
Information content (IC) distribution for the top 30 clusters obtained using Naïve clustering. Upper panels: HPO profile size for each cluster. Lower panels: IC distribution. (**A**) DECIPHER and (**B**) ID/MCA.

This is shown for the the DECIPHER cohort (Figure 5), for which the first four clusters include more than 2500 patients with profile IC values between 0 and 1. These clusters contain patients with profiles describing only one or two HPO terms and frequently contain the same combination of HPO terms repeated for all patients, or possibly including only one different HPO term as shown in Supplementary Table S1. Notably, cluster 10 has an average IC greater than 3.5. The patients within this cluster do often have high IC profiles; however, this is because they have only been phenotyped with a single HPO term, and this HPO term has not been ascribed to any other patients within the database. In fact, this cluster includes 148 different HPO terms described for 148 patients.

In the case of the ID/MCA cohort, clusters include lower number of phenotypes in comparison to the DECIPHER dataset and all members in each cluster have identical phenotypes, except for clusters 6, 17, 22 and 24. These clusters contain patients with profiles containing only one or two HPO terms assigned to all patients within cluster, as shown in Supplementary Table S2. For the PMM2-CDG dataset, the Naïve clustering produces almost as many clusters as patients (data not shown). This is to be expected, given that the patients have been ascribed a large number of phenotypes, with no two patients having the same phenotypic profile.

We conclude that Naïve clustering can identify large groups of patients with very small phenotype profiles (one or two terms per patient) that also have low IC values. These patients do not provide enough information to be used in downstream analysis such as clustering-based semantic similarity to find subgroups of phenotypically similar patients. Consequently, we should consider removing these patients. Patient removal must be performed carefully, since the total number of unique phenotypes used to characterize the cohort can also be affected and some specific phenotypes can be removed. As such, the effects of filtering on the dataset should be examined.

### 3.3. Removing Patients with Limited Phenotypic Information from the Cohort and Its Effect on the Dataset Properties

Given that DECIPHER and ID/MCA datasets contain large numbers of patient with very small phenotype profiles, we investigated the consequences of removing these patients on the summary statistics and other cohort properties.

We see in the rightmost columns of Table 3 that for both datasets, this filter barely reduces the total number of unique HPO terms; however, it reduces the total number of patients in the dataset by almost half. This shows the phenotypes ascribed to the filtered patients were also found among the remaining patients. As expected, the mean HPO terms per patient and HPO terms for percentile 90 both increased. The percentage of HPO terms with more specific child terms only reduces slightly, in line with the low-information patients representing a subset of the terms held by the high-information patients. This shows that removing these patients has little effect on the phenotypic diversity of the dataset. Interestingly, the most common phenotypes actually became more frequent within the DECIPHER and ID/MCA datasets after filtering, suggesting that these phenotypes were more frequently found within longer phenotypic profiles (Supplementary Table S3).

As can be seen in Table 5, for the DECIPHER dataset, the DsI calculated for the unique terms increases very slightly after the filter, showing that the few unique HPO terms that were removed were of lower-information content, this is also reflected by the slight shift to the right in Supplementary Figure S1 compared to Figure 3. However, for weighted terms, the increase was slightly more marked, with the DsI increasing by a larger amount and an appreciable shift towards deeper levels in Supplementary Figure S1. This suggests that the filtered patients not only had few ascribed phenotypes, but that the phenotypes tended to be unspecific. For the ID/MCA dataset, there was no change in DsI—this remained as 0, due to this cohort having HPO terms corresponding to the *High section* levels, something that cannot be improved by anything other than more thorough phenotyping of the patients.

In terms of the IC values (Supplementary Figure S2), we see that for all cohorts, removing the low-information patients has little effect on the distributions of IC values for individual HPO terms, in line with the small reduction in total unique terms across the cohorts (Table 3). However, when the IC values calculated using the phenotypic profiles of each patient are considered, we see a clear smoothing of the distributions, particular for the cohort-frequency calculated values, for both DECIPHER and ID/MCA cohorts. For these datasets, large peaks corresponding to groups of low IC patients are removed, in line with the idea that many of the patients with few phenotypes have also been assigned unspecific ones. For the DECIPHER dataset, there is also a clear peak of high profile IC values for HPO ontology-based values before filtering; this may be due to the patients with single but unique phenotypes found in cluster 10 in Figure 5, the patient IC is the same as the phenotype IC for these patients because their profiles only contain single terms.

This suggests that some of the filtered patients had specific phenotypic profiles according to the ontology, but that were less specific within the cohort itself. No appreciable change is apparent for the PMM2-CDG cohort, unsurprising given that no patients were removed, although the cohort-frequency of the HPO terms changed very slightly.

In terms of the Naïve clustering, for the unfiltered DECIPHER dataset (Figure 5) there were many clusters containing hundreds of patients with identical low IC phenotypes alongside a handful of outlier patients with slightly higher ICs. Removing the very small phenotype patients and repeating the Naïve clustering led to much smaller clusters with a higher range of ICs (Supplementary Figure S3), as would be expected.

This was less clear for the ID/MCA dataset—although several very large clusters were removed, the remaining ones also showed a small range of ICs. This may be due to the patients having fewer phenotypes, most of which had more specific child terms, even after filtering (Table 3), in line with the DsI values of 0 and lower patient level IC values (Supplementary Figure S2), all indicative of these patients having, in general, small phenotypic profiles consisting of unspecific HPO terms.

### 3.4. Comparing Phenotype Profiles to Cluster Patients into Phenotypically-Related Subgroups

After removing poorly-phenotyped patients, it was possible to analyze the cohorts to identify groups of phenotypically related patients. Cohort Analyzer calculates pairwise semantic similarity values between the phenotypic profiles of patients to generate a similarity matrix. Although three distinct similarity measures can be used (Resnik, Lin and Jiang–Conrath), here, we present results for the Lin similarity measure. It normalizes values between 0 (no similarity) and 1 (maximum similarity), allowing the easy calculation of distance matrices for hierarchical clustering.

**Figure 6:**
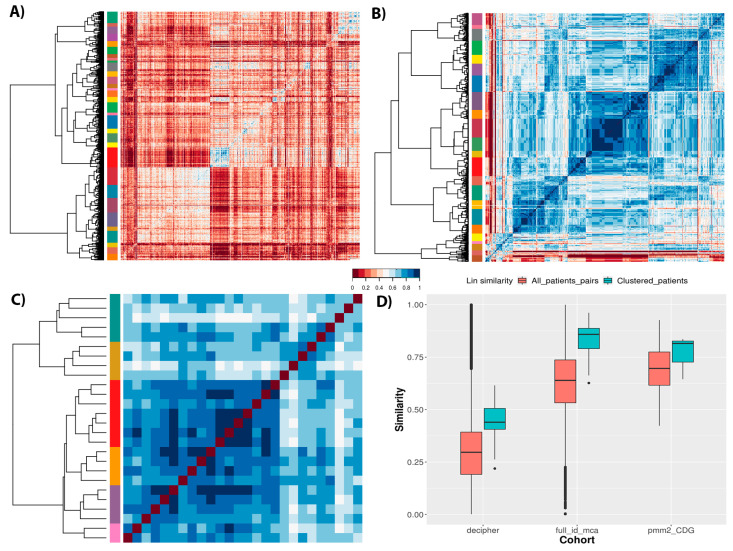
Heatmaps with patient similarity calculated with Lin similarity measure for each pair of profiles. The coloured column shows the patient groups identified by the clustering algorithm. Only patients with three or more HPO terms were used to build the similarity matrix. (**A**) DECIPHER, (**B**) ID/MCA, (**C**) PMM2-CDG. (**D**) Semantic similarity distribution for each cohort along the whole dataset (salmon boxes) or averaged for each cluster (blue boxes).

Figure 6 shows the semantic similarity matrices for the different cohorts, revealing the cohort structure and patient clustering for each. There is clearly much less similarity between most patients within the DECIPHER cohort than the others, in line with the distributions of similarity values for each cohort (Figure 6D, salmon boxes). Notably, both DECIPHER and ID/MCA cohorts show a wide range of similarity values, whilst PMM2-CDG dataset shows a much smaller range, which is unsurprising given the first two are aimed at a wider range of patients, whilst the latter only contains patients diagnosed with the same monogenic disease. It should also be noted that the ID/MCA and PMM2-CDG cohorts have remarkably similar median similarity values (0.63 and 0.69, respectively), despite being very different in most other ways. This highlights the importance of looking at the full distribution of similarity values, and taking into account other cohort-related statistics, rather than simply comparing medians. Returning to the heatmaps, we see clear clusters of similar patients for the different datasets, although it is difficult to compare the datasets directly given the differences in total numbers of patients for each.

Finally, we checked the clustering homogeneity for each cohort calculating the average similarity measure for the members of each patient cluster as shown in Figure 6D, blue boxes. The DECIPHER dataset showed an increase in average similarity to 0.43, suggesting a large number of phenotypically diverse patients per cluster. However, ID/MCA cohort showed the greatest increase average similarity, increasing from a similarity of 0.63 to 0.85. Conversely, PMM2-CDG cohort showed the smallest increment, from 0.69 to 0.81. These results suggest that ID/MCA cohort forms close clusters easily due to the very narrow phenotype spectrum and the small patient profiles, contrary to PMM2-CDG cohort.

### 3.5. Genomic Variant Data Analysis

Cohort Analyzer can also perform analysis of genomic variant data. Firstly, it computes various summary statistics, as shown in Table 6, applied to the three datasets included in this study. We see that variant sizes are much greater for the DECIPHER and ID/MCA datasets; this is because they contain CNV data, whilst the PMM2-CDG dataset contains a range of variants affecting a single gene, as such the variant size refers to the *PMM2* gene coordinates (GRCh37/hg19 human genome assembly). Despite similar variant sizes, the DECIPHER dataset covers a larger proportion of the genome than ID/MCA dataset, in line with it containing a higher number of patients that are more phenotypically distinct.

Cohort Analyzer also includes metrics to analyse the overlap between patient variants. For this, it determines genome windows named Short Overlapping Regions (SOR), which consist of genomic regions shared by at least two patients in a given cohort. In the case of DECIPHER dataset, there are 39,136 genome distinct genomic windows, which are reduced to 39,109 when Cohort Analyzer establishes SORs, i.e., only including regions that overlap between patients. In the case of ID/MCA dataset, there are 1597 genomic windows, of which 1097 can be considered SORs.

With respect to the PMM2-CDG dataset, all metrics present the characteristics of a monogenic disease. Variant size and affected genome nucleotides agree with the *PMM2* gene coordinates and there is only one genome window for all patients.

**Figure 7:**
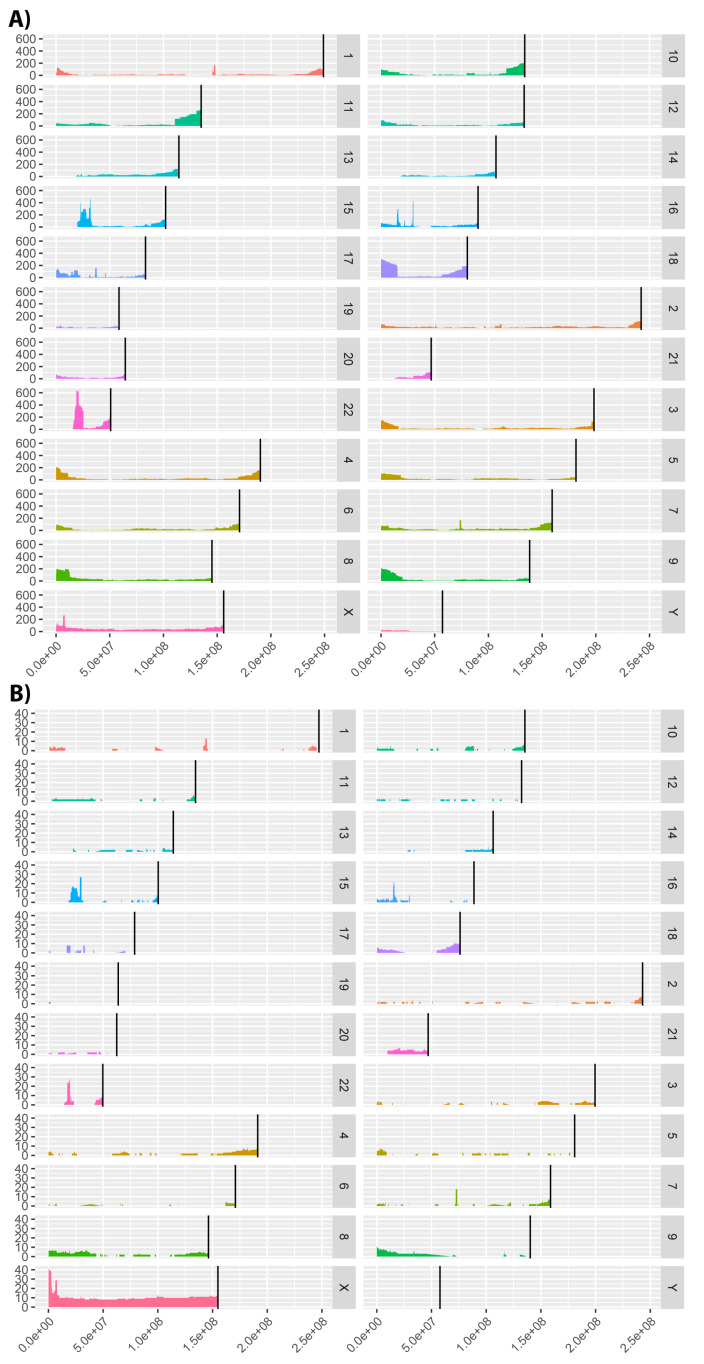
Genome coverage distribution for (**A**) DECIPHER (GRCh38/hg38 human genome assembly) and (**B**) ID/MCA (NCBI36/hg18 human genome assembly). Black vertical bars represent the end coordinate for each chromosome.

Furthermore, Cohort Analyzer generates a genome coverage graph showing patient variant distribution throughout the genome. We show the coverage for the DECIPHER and ID/MCA cohorts in Figure 7. The human genome assembly versions were GRCh38/hg38 and NCBI36/hg18, respectively. Analysis was not performed on the PMM2-CDG dataset as only a single gene locus is implicated in these patients.

The DECIPHER dataset contains patients with variants affecting virtually all of the genome, albeit at low coverage is most places, whilst the ID/MCA dataset shows more defined islands of coverage surrounded by uncovered regions.

Interestingly, there are a number of clear peaks common to both datasets. We analyzed a number of these regions to confirm if they were related to known diseases, using the OMIM [35] and Orphanet [36] databases. Microdeletions in many of these genomic regions are associated with neurological diseases, such as intellectual disability, autism and schizophrenia [37]. Specifically, microdeletions in the 15q11.2 and 16p13.11 regions have been associated with idiopathic generalized epilepsy [37]. Peaks in chromosome 15 are in a genomic region containing variants that have also been associated with Prader–Willi syndrome (15q11-q13 duplication) [38]. Deletions in the 22q11.21-q11.23 region that corresponds to the peak shown in chromosome 22 have been associated with DiGeorge syndrome [39]. This is not as marked in the ID/MCA dataset, consistent with DECIPHER cohort containing more phenotypically diverse patients. In relation to peaks observed for ID/MCA dataset on chromosome X, a large number of diseases involving this chromosome have been described with pathological phenotypes including intellectual disability [40], dystrophinopathies [41] and cardiopathies [42] among others [43].

There are also regions with no coverage in either cohort, for example, the initial base pairs in chromosomes 13, 14, 15, 21 and 22. This may be due to these genomic regions not allowing variation for the viability of the organism, because no patients characterised with mutations in these regions or other limitations. However, more studies are required.

### 3.6. Using Variant Data to Analyse Patient Clusters

**Figure 8:**
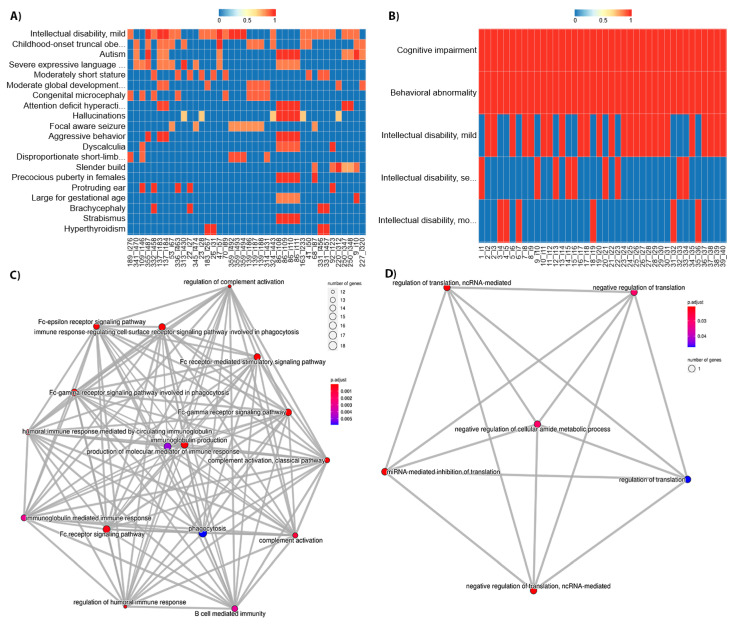
Columns represents DECIPHER and ID/MCA cohorts top semantic similarity cluster examples, respectively. (**A**,**B**) show heatmaps with the semantic similarity between the patients from the cluster and the phenotypic spectra of the cluster itself. The patient axis (x) is sorted according to decreasing semantic similarity with the phenotypic spectra. The HPO axis (y) shows the phenotypic spectra for the cluster, sorted by average semantic similarity with the patient profiles. The top 20 HPO terms and 40 patients are illustrated. (**C**,**D**) show functional enrichment for the genes mapping to the variants shared by patients in the clusters, performed using Gene Ontology, Biological Process terms. Nodes represent GO terms, sized according to the number of patient genes annotated with the given term. The numbers of shared genes between GOs are represented by grey links. The patient identifiers shown in the heatmaps are randomly generated and bear no relation to the original cohort identifiers.

The variant information allows us to infer which genes are affected in the patients. We can use this information combined with the patient subgroups generated by semantic clustering to identify functional systems potentially related to the phenotypes for each. As a case study, we selected the patient subgroups from the DECIPHER and ID/MCA datasets with highest average similarity to show how the patient phenotypes relate to the functional systems that the affected genes are involved in. For this, we first obtained a phenotypic spectrum for the cluster itself, and then we calculated the semantic similarity between each HPO term in each of the patient profiles with those of the phenotypic spectrum. This allowed us to see which phenotypes are representative of each cluster, as they will show high similarity for most of the patients (Figure 8, upper panels). These representative phenotypes can then be related with the functional analysis results (Figure 8, bottom panels). For DECIPHER dataset (Figure 8A), the phenotypes are rather heterogeneous and similarity is not regularly distributed across all patients. On the contrary, for the ID/MCA dataset cluster (Figure 8B) the phenotypic spectra is shared by all the patients, although one of them (HP: “Intellectual disability”) is split into grades. This phenotypic spectra is very general compared to the DECIPHER dataset spectra, in agreement with the functional analysis results; the ID/MCA dataset cluster only shows protein translation-related categories (Figure 8D) whereas the DECIPHER dataset cluster shows a range of categories related to immune system including phagocytosis, complement activation and humoral immune response mediated by circulating immunoglobulin (Figure 8C). Previous studies have suggested a genetic link between the immune system and several top phenotypes for this cluster including intellectual disability [44], obesity [45] and autism [46].

## 4. Discussion

Deep phenotyping is essential to understanding and diagnosing genetic diseases [47]. As such, using a standardized vocabulary of terms, such as the HPO [25], is crucial for consistent phenotyping. Among its multiple applications, the HPO has been used to develop different tools for guiding diagnosis [12,13,48] and to understand disease mechanisms [10,49]. However, nothing currently exists to assess the overall quality of a cohort annotated using the HPO. We have developed Cohort Analyzer to address this problem. It can be used to analyse any patient cohort for which phenotypic data is available in the form of HPO terms, and optionally genomic data in the form of genome coordinates. Here, we have applied it to vastly different patient cohorts.

More specific terms are linked to higher quality phenotyping [50,51]. Based on this assumption, we have proposed a new measure, the DsI, to assess phenotype specificity for a cohort. Applying it to the three cohorts allowed us to show that the PMM2-CDG patients were phenotyped to a greater depth than the other cohorts. This finding was reinforced by many of the other statistics and plots produced by Cohort Analyser; the patients tended to have greater numbers of terms and these terms were less likely to have more specific child terms. This is strikingly apparent in Figure 2. The DECIPHER cohort led to a higher DsI than the ID/MCA cohort, this is unsurprising given that none of the patients in the latter had been ascribed phenotypes deeper than level 8 in the HPO and also fits with the very high number of HPO terms with more specific child terms for the patients in this cohort.

Whilst the DSI gives a clear indication of phenotyping depth, it should be interpreted alongside other summary metrics and plots, such as the “Percentage of HPO terms with more specific child terms”, as well as the nature of the analyzed cohort. This is because some diseases are underrepresented phenotypically in the HPO, such as respiratory disorders [52] and phenotypes related to these diseases may not reach deeper levels.

The PMM2-CDG dataset covers a small number of patients with a severe and rare monogenic disease. These patients have been followed up extensively and this is clear in the results of applying Cohort Analyzer. Deep phenotyping is crucial to better understand this disease and distinguish patients from each other to identify subgroups and patterns. On the other hand, both DECIPHER and ID/MCA cohorts cover a much wider range of patients in terms of both phenotypes and genetic causes.

We have also shown the importance of a minimum phenotype profile length. Many patients in the DECIPHER and ID/MCA datasets were described with a single HPO term. Though these were generally unspecific, there were some exceptions in the DECIPHER cohort, such as patients ascribed only HP: “Median cleft lip and palate”, at the 13th HPO level. According to Orphanet, this phenotype has been frequently associated with different genes and described in many diseases, such as Loeys–Dietz syndrome [53] and the autosomal dominant Robinow syndrome [54], both of which occur alongside other phenotypes. As such, this phenotype on its own is insufficient to either aid diagnosis or help interpret the genetic variants for this patient.

The ID/MCA cohort is described as a group of patients with both intellectual disability/developmental delay (ID/DD) and multiple congenital anomalies (MCA) [24]. These pathologies co-occur alongside multiple additional phenotypes, as such the cohort can be considered phenotypically heterogeneous, such as DECIPHER. Nevertheless, two thirds of the ID/MCA patients were described with two or fewer phenotypes. Combined with the low specificity of phenotypes in this cohort, as shown by its DsI of 0, it suggests that some of these patients may actually suffer from additional phenotypes that they have not been assigned. Most of these patients were described with HP: “Cognitive impairment”, a pathological phenotype that frequently appears related to patients with ID [55,56] and MCA [57,58]. This term actually has 26 descendant terms within the HPO hierarchy. DECIPHER cohort also had a large number of patients ascribed unspecific terms with multiple descendants, as shown in the Naïve clustering analysis (Figure 5). However, the filtering of patients with fewer than three phenotypes removed many of these patients, and this improved the dataset in terms of the DsI and other parameters. This filtering step was particularly important for the next stages of the analysis, looking for subgroups of phenotypically similar patients and relating phenotype information to the genomic variants of these patients. Interestingly, the semantic similarity clustering to find patient subgroups led to very similar clusters for the ID/MCA group, however it is likely that this similarity is due to the patients having small and very general phenotypes, and tempting to speculate that deeper phenotyping of these patients might reveal difference within the clusters.

In terms of the cohort-wide variant coverage, the ID/MCA and DECIPHER datasets showed multiple shared peaks, in line with previous studies showing that some of common phenotypes in the cohorts can be caused by a range of variants [59,60,61]. This suggests that the pathologies of these patients may have overlapping genomic causes. A such, we might expect more similar phenotypic profiles, however, as stated before, the DECIPHER patients tended to be assigned a greater number of deeper phenotypes. This has an important impact on the ability to link phenotypes with genes and functions based on shared variants between phenotypically similar patients, as shown by the stark contrast between the results in Figure 8.

There may be other reasons for these overlapping peaks, and it should be made clear that these peaks, as well as the summary statistics related to the genomic data, should be interpreted in terms of the technology used and type and size of variant being investigated. The use of variant data from control groups such as individuals from the 1000 genomes project [62], or structural data from resources such as the Database of Genomic Variants [63] could be analyzed alongside the patient cohort, to look for differences. Moreover, tools such as CNVxplorer could be used to help locate specific regions of interest [64].

One way to improve the phenotyping of patients would be to review each of the cohorts and apply additional diagnostic tests to find more specific terms. An important consideration to keep in mind regarding phenotyping of patients is that they evolve with the patient; a child up to five years can be classified with HP: “Global developmental delay”, but from this age it will be classified with HP: “Intellectual disability”. This can potentially lead to a less informative phenotypic profile if not accompanied by additional phenotypes. Therefore, evaluating the evolution that the phenotypes follow could also be an important factor when characterizing patients and should be taken into account when assessing phenotyping quality. This can also happen with certain organs that stabilize over time, such as some liver disorders [65] and protein-losing enteropathy [66]. These pathologies can improve over time and disappear. However, their relationship with the genotype depends on whether or not it was ever in the patient history. Dysmorphic features can also change over time [33]. For this reason, children are mostly annotated with phenotypes such as HP: “Retrognathia”, but when they become older are re-annotated with HP: “Mandibular prognathia”. Interestingly, the most common phenotypes in the PMM2-CDG cohort, aside from HP: “Cerebellar Atrophy”, are largely related to dysmorphism. These can complement more classic disease phenotypes such as those related to neurodevelopment. Further work could investigate the subgroups for this cohort to see how the different dysmorphic features cluster with other phenotypes. This can give additional insights into the disease and help orientate future study.

A limitation of the tool is that patients must be phenotyped using HPO terms. However, as text mining analysis of electronical health records (EHR) improves and becomes more commonplace, this is likely to become less of a barrier. In fact, our tool could potentially be used to aid the development of these techniques. Nowadays there is a strong research effort focused on translating EHR to standardized vocabularies, with multiple potential applications for patient diagnosis and treatment. An important approach in this area is the use of machine learning and Natural Language Processing (NLP) techniques to convert EHR into HPO profiles [67]. Our tool could measure the performance of these techniques and thus allow researchers to optimize their methodology.

To conclude, this work highlights the need for improved phenotyping in this era of personalized medicine. We have shown that Cohort Analyzer can help in achieving this goal, by providing a tool for the analysis of the phenotypic quality of patient cohorts, identifying generalised problems, sets of patients that could be re-examined, subgroups of phenotypically similar patients, and relating this information to genomic variant data to suggest affected underlying functions.

## Figures and Tables

**Table 1:** Cohort Analyzer general summary statistics.

Name	Description
Unique HPO terms	Number of distinct HPO terms that are used to describe the patients in the cohort
Cohort size	Number of patients in the cohort.
HPO terms per patient (average)	Mean number of HPO terms per patient in the cohort.
HPO terms per patient: percentile 90	Sorting patients from the highest to the lowest phenotype profile length, the number of HPO terms of the patient positioned at percentile 90 in the list. This gives an idea about the amount of phenotypic information available for the least well phenotyped members of the cohort.
Percentage of HPO terms with more specific child terms	The percentage of phenotypes assigned to patients in the cohort for which more specific terms are available within the HPO to characterize the patient.
Average variant size	Average length in base pairs of all variants belonging to patients within the cohort.
Nucleotides affected by mutations	Total number of base pairs covered by variants belonging to patients within the cohort.
Number of genome windows	Distinct contiguous genomic regions corresponding to segments of variants within the cohort, segmented such that each region belongs to a distinct combination of patients.
Number of genome window shared by ≥ 2 patients	The number of affected genome windows shared by at least two patients, also referred to as short overlapping regions.
Mean patients per genome window	Average number of patients that correspond to the genomic regions within the cohort.

**Table 2:** General characteristics related to the three datasets used in this study. For the DECIPHER dataset, gender information was not available for the subset of patients used in this analysis, however this information can be viewed for specific patients on the DECIPHER website.

	PMM2-CDG	ID/MCA	DECIPHER
Total Patients	27 with HPO annotation and genotype data	4183 with HPO annotation; 1027 with HPO and genotype data	22,018 with HPO annotation; X with HPO and genotype data
Age (median; IQR)	12 (9–17)	7 (3–17)	NA
Gender:(% female)	44.4%	41.9%	NA
Diseases covered	PMM2-congenital disorder of glycosylation	Multiple congenital anomalies-intellectual disability	Range of heterogeneous complex diseases

**Table 3:** Cohort Analyzer general summary statistics. Results for the datasets following filtering to remove patients with less than three assigned HPO terms are indicated with >2.

Name	DECIPHER	ID/MCA	PMM2-CDG	DECIPHER > 2	ID/MCA > 2
Unique HPO terms	3670	24	62	3481	24
Cohort size	22,018	3971	27	12,044	1932
HPO terms per patient (average)	5.36	2.71	25.25	8.59	4.03
HPO terms per patient: percentile 90	1	1	15	3	3
Percentage of HPO terms with more specific child terms	63.36	81.71	52.78	61.36	78.65

**Table 4:** Top five most frequent HP0 terms in DECIPHER, ID/MCA and PMM2-CDG datasets.

	DECIPHER	%	ID/MCA	%	PMM2-CDG	%
1	Intellectual disability	34.98	Cognitive impairment	80.96	Cerebellar atrophy	100.00
2	Global developmental delay	14.20	Intellectual disability, mild	17.67	Upslanted palpebral fissure	88.88
3	Delayed speech and language development	12.87	Short stature	17.32	High, narrow palate	85.18
4	Microcephaly	9.20	Autistic behavior	16.87	Strabismus	81.48
5	Hypotonia	8.48	Hypotonia	15.36	Anteverted nares	74.07

**Table 5:** Dataset specificity Index for each cohort. Results for the datasets following filtering to remove patients with less than three assigned HPO terms are indicated with >2.

Frequency	DECIPHER	ID/MCA	PMM2-CDG	DECIPHER > 2	ID/MCA > 2
Unique	0.13	0.00	0.27	0.19	0.00
Weighted	0.19	0.00	1.06	0.31	0.00

**Table 6:** Cohort Analyzer general summary statistics. The information listed in this table refers to the patients phenotyped with at least three HPO terms and with characterized genomic variants.

Name	DECIPHER	ID/MCA	PMM2-CDG
Average variant size	5,053,537.45	3,498,347.16	51,524.00
Nucleotides affected by mutations	2,917,478,733	1,266,576,677	51,524
Number of genome windows	21,578	860	1
Number of genome window shared by ≥2 patients	21,522	466	1
Mean patients per genome window	48.59	2.49	27.00

## Data Availability

The datasets used and/or analyzed during the current study are available from the DECIPHER database under signed agreement. All code underlying the Cohort Analyzer tool is freely available from https://github.com/ElenaRojano/pets, written in Ruby, and code underlying the workflow is available at https://github.com/JoseCorCab/cohortAnalyzer_wf. This workflow uses the manager AutoFlow and can be run on UNIX-like systems. All dependencies are explained in the README file of the GitHub repository.

## Supplementary Materials

The following are available online at https://www.mdpi.com/article/10.3390/jpm11080730/s1, Supplementary Table S1: Topfive patient clusters from the DECIPHER cohort using the Naïve method, Supplementary Table S2: Top five patient clusters from the ID/MCA cohort using the Naïve method, Supplementary Table S3: Top five most frequent HPO terms in the DECIPHER, ID/MCA and PMM2-CDG datasets, Supplementary Figure S1: HPO terms percentage distribution plots for (A) DECIPHER, (B) ID/MCA and (C) PMM2-CDG cohorts, Supplementary Figure S2: Information content (IC) distribution for (A) DECIPHER, (B) ID/MCA and (C) PMM2-CDG cohorts, Supplementary Figure S3: Information content (IC) distribution for the top 30 clusters calculated with Cohort Analyzer.

## Abbreviations

The following abbreviations are used in this manuscript:

aCGH	Microarray-based Comparative Genomic Hybridization
CNV	Copy Number Variant
DD	Developmental Delay
DsI	Dataset specificity Index
EHR	Electronical Health Records
GO	Gene Ontology
HPO	Human Phenotype Ontology
HsS	High section Score
IC	Information Content
ID	Intellectual Disability
LsS	Low section Score
MCA	Multiple Congenital Diseases
NLP	Natural Language Processing
PMM2-CDG	Phosphomannomutase 2 Congenital Disorder of Glycosylation
SOR	Short Overlapping Region
