# Supplementary material for: Evaluating, Filtering and Clustering Genetic Disease Cohorts Based on Human Phenotype Ontology Data with Cohort Analyzer
Source: J Pers Med. 2021 Jul 27;11(8):730. doi: 10.3390/jpm11080730 (PMC8398478; doi:10.3390/jpm11080730)
Supplement: Supplementary file 1 [file jpm-11-00730-s001.zip › jpm-1269477-supplementary.pdf]

Clust. ID	Pats.	Phenotypes	Frequency
0	1225	Intellectual disability, Turricephaly, Abnormality of the palpebral fissures, Elbow dislocation, Neoplasm of the gastrointestinal tract, Cerebral atrophy, Narrow mouth, Abnormality of the seventh cranial nerve, Round face, Glaucoma, Increased serum lactate, Bruxism, Long eyelashes, Scleroderma, Achilles tendon contracture, Renal cyst, Episodic ataxia, EEG with localized low amplitude activity, Meningioma, Psychotic mentation, Bilateral cleft lip and palate, 2-3 finger syndactyly, Hepatoblastoma, Abnormality of the thyroid gland, Early onset of sexual maturation, Abnormal hair pattern, Conspicuously happy disposition, Abnormal form of the vertebral bodies, Stroke, Age-related cataract, Metatarsus adductus, Abnormality of body weight, Distal shortening of limbs, 2-4 toe cutaneous syndactyly, Gonadal dysgenesis with female appearance, male, Cutaneous photosensitivity, Radioulnar synostosis, Retinal dystrophy, Abnormality of connective tissue, Protruding tongue, Atonic seizure, Abnormal shape of the frontal region, Rhabdomyolysis, Nonprogressive cerebellar ataxia, Immunodeficiency, Complete duplication of thumb phalanx, Inferior vermis hypoplasia, Cerebellar malformation, Diffuse white matter abnormalities, Limb tremor, Macroorchidism, Myelomeningocele, Unilateral cryptorchidism, Abnormality of the cerebral vasculature, Precocious puberty in males, Multiple cafe-au-lait spots, Chronic kidney disease, Pseudohypoaldosteronism	100.0, 0.0816
1	738	Global developmental delay, Bulimia, Abnormality of the cerebellar vermis, Delayed peripheral myelination, Esotropia, Cystic renal dysplasia, Cerebellar malformation, Bilateral cleft palate, Weight loss, Communicating hydrocephalus, Frontoparietal polymicrogyria, Vesicoureteral reflux, Impulsivity, Myopia, Oligodontia, Abnormal pulmonary valve morphology, Encephalitis, Secundum atrial septal defect, Cerebral hamartoma, Generalized joint laxity, Early onset of sexual maturation, Submucous cleft soft palate, Lower limb hyperreflexia, Amenorrhea, Patellar aplasia, Highly arched eyebrow, Chronic otitis media, Abnormal facial expression, Gait disturbance, Hip dysplasia, Excessive salivation, Chronic constipation, Happy demeanor, Finger syndactyly, Dandy-Walker malformation, Neonatal inspiratory stridor, Cerebral ischemia, Bilateral cleft lip and palate, High myopia, Hashimoto thyroiditis, Profound hearing impairment, Inflexible adherence to routines or rituals, Monocular strabismus, Syncope, Coronal craniosynostosis, Cerebellar dysplasia, Progressive language deterioration, Abnormal cerebellum morphology, Hand polydactyly	100.0, 0.1355
2	342	Aplasia/Hypoplasia of the thymus, Autism, Sacral dimple, Long palpebral fissure, Tics, Coarse facial features, Epicanthus, Hearing abnormality, Muscle weakness, Obsessive-compulsive behavior, Conductive hearing impairment, Increased serum lactate, Loss of speech, Personality disorder, Abnormality of bone marrow cell morphology, Vesicoureteral reflux, Hypopigmented skin patches, Premature adrenarche, Psychotic episodes	0.2924, 100.0, 0.2924, 0.2924, 0.2924, 0.2924, 0.2924, 0.2924, 0.2924, 0.2924, 0.2924, 0.2924, 0.2924, 0.2924, 0.2924, 0.2924, 0.2924, 0.2924, 0.2924
3	259	Autistic behavior, Hypopigmented skin patches on arms, Esotropia, Monocular strabismus, Headache, Visual impairment, Downturned corners of mouth, Chronic constipation, Postural instability, Myopia	100.0, 0.3861, 0.3861, 0.3861, 0.3861, 0.3861, 0.3861, 0.3861, 0.3861, 0.3861
4	255	Cognitive impairment, Sleep apnea, Dandy-Walker malformation, High pitched voice, Truncal obesity, Abnormal cerebellum morphology, Cerebral calcification, Cryptorchidism, Pes planus, Bifid uvula, Pseudohypoparathyroidism, Type E brachydactyly	100.0, 0.3922, 0.3922, 0.3922, 0.3922, 0.3922, 0.3922, 0.3922, 0.3922, 0.3922, 0.3922, 0.3922

Supplementary Table S1: Top five patient clusters from the DECIPHER cohort using the Naïve method, including HPO terms and their frequencies for each cluster

Supplementary Table S2: Top five patient clusters from the ID/MCA cohort using the Naïve method, including HPO terms and their frequencies for each cluster

**Cluster ID**	**Patients**	**Phenotypes**	**Frequency (%)**
0	688	Cognitive impairment	100.0
1	137	Cognitive impairment, Intellectual disability, mild	100.0, 100.0
2	106	Cognitive impairment, Autistic behavior	100.0, 100.0
3	259	Cognitive impairment, Behavioral abnormality	100.0, 100.0
4	255	Cognitive impairment, Intellectual disability, moderate	100.0, 100.0

Supplementary Table S3: Top five most frequent HPO terms in the DECIPHER, ID/MCA and PMM2-CDG datasets. Patients with two or less HPO terms in their profile were removed

	**DECIPHER**	%	**ID/MCA**	%	**PMM2-CDG**	%
1	Intellectual disability	42.593	Cognitive impairment	93.426	Cerebellar atrophy	100.0
2	Delayed speech and language development	18.872	Intellectual disability, mild	29.244	Upslanted palpebral fissure	88.888
3	Microcephaly	14.754	Intellectual disability, severe	28.933	High, narrow palate	85.185
4	Hypotonia	14.621	Short stature	27.173	Strabismus	81.481
5	Global developmental delay	14.031	Seizure	26.501	Anteverted nares	74.074

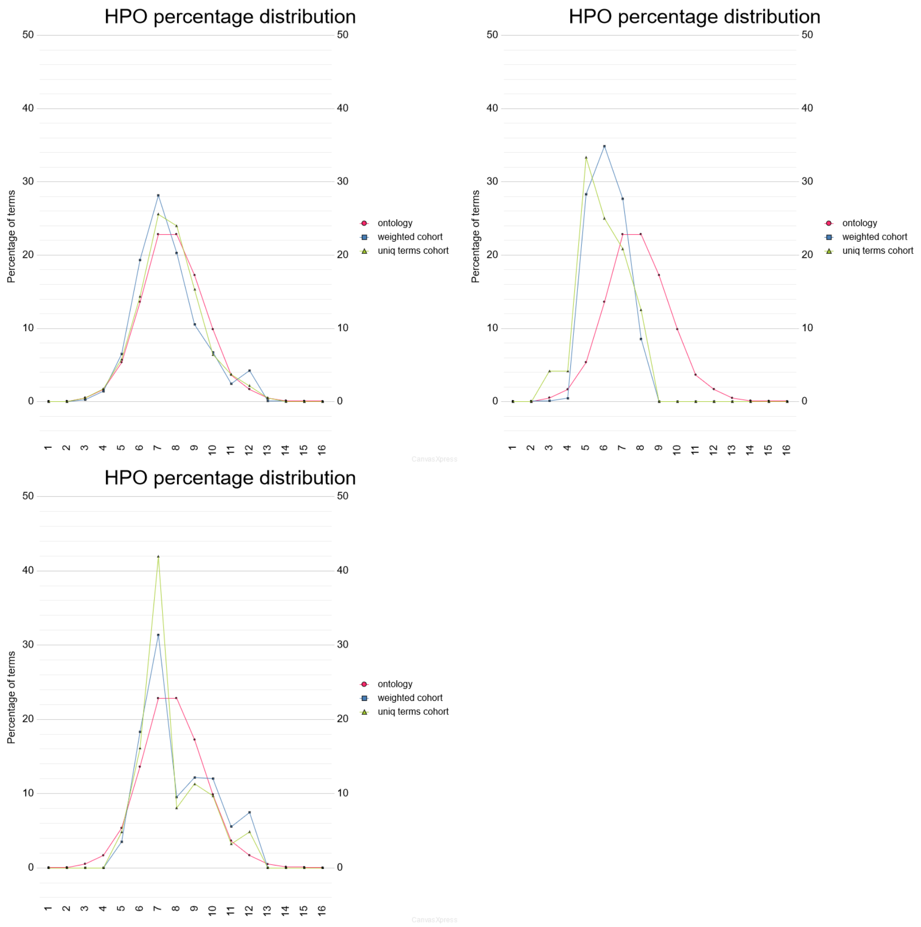

Supplementary Figure S1: HPO terms percentage distribution plots for A) DECIPHER, B)ID/MCA and C) PMM2-CDG cohorts. Green curves represent unique HPO terms used to describe the cohort, blue curves represent the frequency of each term and pink curves are the number of terms included in each HPO level. Patients with two or less HPO terms in their profile were removed from cohort.

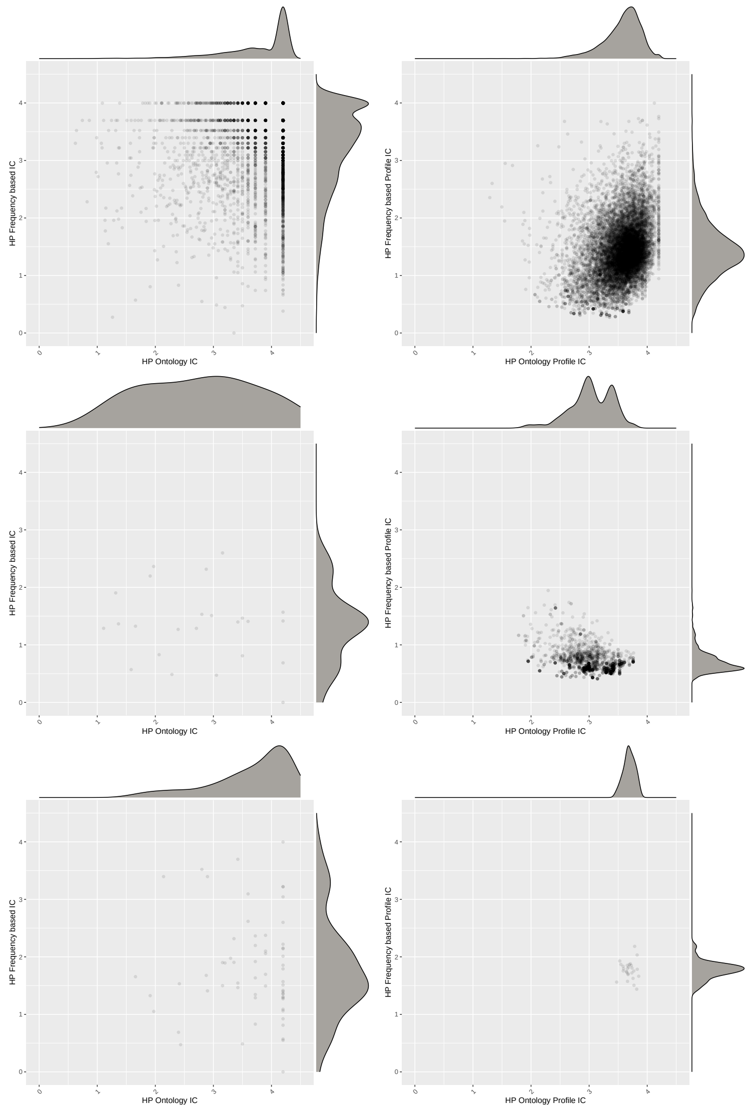

Supplementary Figure S2: Information content (IC) distribution for A) DECIPHER, B)ID/MCA and C) PMM2-CDG cohorts. Left figures correspond to HPO terms within HPO and right figures to phenotype profiles in each cohort. To compare the cohorts a general IC table was computed for the "Frequency based IC" axis with all patients used in the three cohorts with at least three assigned HPO terms. Patients with two or less HPO terms in their profile were removed from cohort.

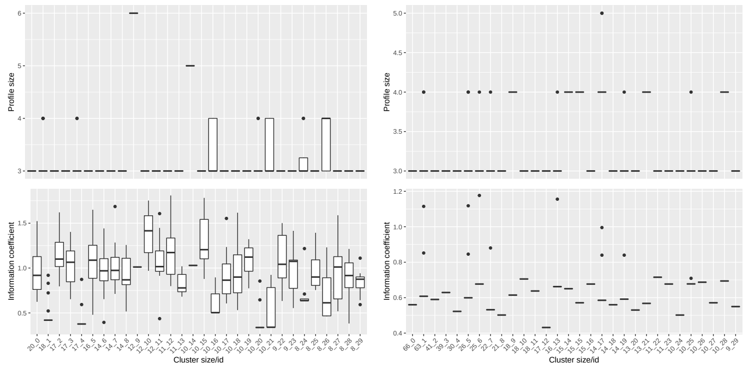

Supplementary Figure S3: Information content (IC) distribution for the top 30 clusters calculated with Cohort Analyzer. Upper figures: HPO profile size for each cluster. Lower figures: IC distribution. A) DECIPHER and B)ID/MCA . Patients with two or less HPO terms in their profile were removed from cohort.
